# Sex differences in genotype frequency and the risk of polycythemia associated with rs13419896 and rs2790859 among Tibetan highlanders living in Tsarang, Mustang, Nepal

**DOI:** 10.1186/s40101-024-00372-5

**Published:** 2024-10-15

**Authors:** Hiroaki Arima, Takayuki Nishimura, Sweta Koirala, Masayuki Nakano, Hiromu Ito, Tomo Ichikawa, Kishor Pandey, Basu Dev Pandey, Taro Yamamoto

**Affiliations:** 1 Department of International Health and Medical Anthropology, Institute of Tropical Medicine, Nagasaki University, 1-12-4 Sakamoto, Nagasaki, 852-8523 Japan; 2 Department of Human Life Design and Science, Faculty of Design, Kyusyu University, 4-9-1 Shiobaru, Minami-Ku, Fukuoka, 815-8540 Japan; 3 Nepal Development Society, Ward 29, Naubise, Kaski District, Pokhara Metropolitan City, Nepal; 4 Department of Nutrition, Faculty of Health Sciences, Kochi Gakuen University, 292-26 Asahitenjin-Cho, Kochi, 780-0955 Japan; 5 Department of Society and Regional Culture, Okinawa International University, 2-6-1 Ginowan, Okinawa, 901-2701 Japan; 6 Central Department of Zoology, Tribhuvan University, Kirtipur, Nepal; 7 DEJIMA Infectious Disease Research Alliance, Nagasaki University, 1-12-4 Sakamoto, Nagasaki, 852-8523 Japan; 8 General Medicine, Medical Office, Saku City Asama General Hospital, 1862-1 Iwamurada, Saku, Nagano, 385-8558 Japan

**Keywords:** Sex difference, Tibetan highlander, Polycythemia, *EGLN1*, Mustang

## Abstract

**Background:**

Tibetan highlanders have adapted to hypoxic environments through genetic mechanisms that avoid hemoglobin concentration increases and prevent polycythemia. Recently, sex differences in hemoglobin dynamics with age have been reported among Tibetan highlanders living in Tsarang. Additionally, concerns have been raised that dietary changes associated with modernization may increase the risk of polycythemia and lifestyle-related diseases among Tibetan highlanders. However, the relationship between genetic polymorphisms and the risk of lifestyle-related diseases in Tibetan highlanders has been investigated in only a few regions. This study aims to elucidate whether polymorphisms in genes related to hypoxic adaptation are associated with the incidence of lifestyle-related diseases and polycythemia and whether these polymorphisms affect hemoglobin dynamics in the residents of Tsarang, Mustang, Nepal.

**Methods:**

Health checkup data from individuals living in Tsarang in Mustang District, Nepal, collected in 2017, were used to determine the prevalence of obesity, hypertension, diabetes, hypoxemia, and polycythemia. DNA was extracted from whole-blood samples, and data for the single-nucleotide polymorphisms (SNPs) rs13419896 (*EPAS1*), rs12619696 (*EPAS1*), and rs2790859 (*EGLN1*) were obtained using real-time PCR. The health checkup data were statistically analyzed to determine the associations of these diseases with polymorphisms in genes related to hypoxic adaptation.

**Results:**

A total of 168 participants, comprising 78 males and 90 females, were included in the final analysis. In terms of the prevalence of each disease, only the prevalence of polycythemia significantly differed between sexes (*p* < 0.01). Additionally, among the three analyzed SNPs, significant sex differences in genotype frequency were observed for rs13419896 and rs2790859. For rs2790859 in females, Tibetan highlanders with the adaptive genotype had a significantly lower incidence of polycythemia (*p* < 0.01) and significantly lower hemoglobin concentrations (*p* < 0.01).

**Conclusions:**

This study revealed that there are sex differences in the genotype frequency of gene-related hypoxic adaptations among the residents of Tsarang. The findings also suggested that the rs2790859 polymorphism might be involved in the recent incidence of polycythemia among Tsarang residents. If the frequency of non-Tibetan genotypes increases due to intermixing with other populations in the Mustang District, polycythemia may emerge as a modern disease. It is essential to continue investigating the health status of Mustang residents to elucidate various aspects of hypoxic adaptation and disease susceptibility.

## Background

The human body is equipped with a mechanism to detect decreases in blood oxygen levels. The carotid bodies, located in the carotid arteries, function as peripheral chemoreceptors, sensing changes in the partial pressure of arterial oxygen and pH in the blood [1]. Additionally, the carotid bodies are aggregates of glomus cells that are in contact with blood vessels and nerve fibers, and these cells are involved in the rapid cardiopulmonary reflexes triggered by hypoxia [2]. On the other hand, mechanisms by which cells respond to hypoxia through cellular metabolism have also been revealed. When blood oxygen levels decrease, the degradation of hypoxia-inducible factors (HIFs) by prolyl hydroxylase 2 (PHD2), which typically occurs under normoxic conditions, is inhibited. As a result, a response to hypoxia that is mediated by HIFs is initiated. This response leads to increased production of erythropoietin and angiogenesis, among other processes, thereby increasing oxygen circulation in the body to adapt to hypoxic environments [3].

Since the early 1900s, anthropological and physiological studies have been performed to determine whether humans can genetically adapt to high-altitude (above 2500 m) environments. This specific altitude cutoff is used because arterial blood oxygen saturation levels begin to decrease in most individuals at 2500 m [4]. Approximately 140 million people worldwide currently live at altitudes above 2500 m. Among them, populations in Tibet, the Andes, and Ethiopia are believed to have genetically adapted to low oxygen levels over many generations [5]. Tibetan highlanders, who have been living in harsh hypoxic environments for generations, have developed adaptations to increase their oxygen circulation. Tibetan highlanders maintain hemoglobin (Hb) concentrations similar to those of lowland dwellers while having increased nitric oxide (NO) concentrations in their blood to dilate blood vessels [6]. Compared with other highlander populations, Tibetan highlanders have single-nucleotide polymorphisms (SNPs) within genes such as *EPAS1* and *EGLN1*, which are involved in the hypoxic response, and these SNPs exhibit significantly different genotype frequencies. This variation enables unique adaptations in Tibetan highlanders, which are distinct from the increase in Hb concentration, allowing them to adapt to hypoxic environments effectively [7, 8]. This hypoxic adaptation mechanism could mitigate the cardiovascular disease risk associated with polycythemia (chronic mountain sickness) observed in Andean highlanders [9, 10].

In a recent epidemiological study of Tibetan highlanders living in Tsarang, Nepal, which is located at an altitude of 3560 m, 10.8% of females were classified as having polycythemia [11]. Furthermore, while it is generally known that the Hb concentration decreases with age [12], the Hb concentration in Tsarang females tends to increase with age [13]. Aging and lifestyle changes among modern Tibetan highlanders may contribute to an increased incidence of diabetes [14]. Indeed, residents of Tsarang have a greater prevalence of prediabetes than the lowland Nepalese population [11]. Furthermore, previous studies of individuals living in Qinghai and Ladakh have indicated the possibility that lifestyle diseases induced by dietary changes further lead to vascular deterioration and metabolic disorders, potentially triggering polycythemia due to decreased oxygen circulation efficiency [15].

Specifically, differences in the genotype frequencies of rs13419896 and rs12619696 in *EPAS1* between Han Chinese and Tibetan highlanders have been reported. These polymorphisms are implicated in the risk of chronic mountain sickness and the hypoxic adaptation observed in Tibetan highlanders and Sherpa populations [16–18]. Tibetan highlanders presented genotype frequencies in EGLN1 that differed from those of the Han Chinese, Japanese, European, and African populations. The rs2790859 polymorphism has been suggested to be associated with the risk of acute mountain sickness [19, 20]. The genotypes associated with this hypoxic adaptation and chronic mountain sickness may also be related to lifestyle disease risk and specific Hb dynamics in females among the residents of Tsarang. However, this relationship has not been investigated in Tibetan highlanders in Nepal. In this study, we attempted to analyze the genotypes of SNPs previously implicated in hypoxic adaptation and chronic mountain sickness among samples from residents of Tsarang. We aimed to clarify whether these genotypes are associated with the prevalence of lifestyle diseases and polycythemia and the specific Hb dynamics observed uniquely in Tsarang.

## Methods

### Population characteristics

This study utilized data and samples collected from participants in a cross-sectional epidemiological study conducted in 2017 in Tsarang village, Mustang district, Western Nepal. Tsarang is situated at an altitude of 3560 m. The total population of Tsarang was reported to be 452 individuals residing in 132 households. Among them, 179 males and 190 females were above the age of 18 years [21]. In 2017, 85 males and 103 females aged 18 years and older participated in the health survey. For this study, data and samples from 78 males and 90 females who provided blood samples were ultimately used. The mean age of the participants was 45.0 ± 13.1 years for males and 48.2 ± 15.5 years for females.

### Acquisition of physiological data

We acquired data such as age, sex, height, weight, systolic and diastolic blood pressure (OMRON Model, HEM-7210, Kyoto, Japan), Hb concentration (ASTRIM FIT health monitoring analyzer: Sysmex, Kobe, Japan), saturation of peripheral oxygen (SpO_2_; pulse oximeter: Masimo Radical V 5.0, Masimo Corp, CA, USA), and glycated hemoglobin (HbA1c; DCA Vantage analyzer, Siemens Healthcare Diagnostics, Munich, Germany) from an epidemiological study that was conducted in Tsarang, Mustang District, in 2017 [11]. Height was measured using a wall-mounted tape measure at the Tsarang health examination site, with the measurement taken at the point where the top of the participant’s head aligned with the tape. Weight was measured using an analog scale.

### Definition of study variables

Body mass index (BMI) (kg/m^2^) was calculated from height and weight, and participants with a BMI > 25 were classified as obese. Participants with systolic blood pressure ≥ 140 mmHg or diastolic blood pressure ≥ 90 mmHg were categorized as having hypertension. Male participants with Hb concentrations ≥ 18 g/dL and female participants with Hb concentrations ≥ 16 g/dL were classified as having polycythemia. An SpO_2_ < 90% was defined as hypoxemia, and an HbA1c ≥ 6.5% was considered indicative of diabetes mellitus.

### Genotyping

We extracted DNA from whole-blood samples using the DNA Blood Mini Kit (Qiagen, Hilden, Germany) and detected the target SNP variants using real-time PCR performed with a LightCycler 480 (Table 1) [22–24]. The reaction conditions were set as follows: a preincubation step at 95 °C for 10 min, followed by 40 cycles of amplification consisting of denaturation at 95 °C for 10 s, annealing at 60 °C for 1 min, and extension at 72 °C for 1 s. The reaction was terminated with a cooling step at 40 °C for 30 s [18, 20, 25, 26]. In this study, we included 78 male and 90 female participants who underwent genotyping of blood samples as the final study population.

**Table 1:** Target alleles

ID	Gene	Chromosome	rs number	Alleles	Population	Allele ratio (A:G or C:T)	
1	*EPAS1*	2p21	rs13419896	A/G	EAS	0.3046	0.6954
					SAS	0.2035	0.7965
2	*EPAS1*	2p21	rs12619696	A/G	EAS	0.4970	0.5030
					SAS	0.1708	0.8292
3	*EGLN1*	1q42	rs2790859	C/T	EAS	0.4534	0.5466
					SAS	0.5276	0.4724

The ancestral alleles are G in rs13419896, G in rs12619696, and T in rs2790859. Among Tibetan highlanders, A in rs13419896, A in rs12619696, and T in rs2790859 were dominant in a previous repor

*EAS* East Asian, *SAS* South Asian

### Statistical analysis

First, the physiological data of the subjects were descriptively summarized as the mean ± SD and the number of subjects (%) exceeding the reference values for each physiological parameter. Group comparisons for continuous variables were conducted using Welch’s *t* test, whereas comparisons of proportions between groups were performed using the chi-square test, Fisher’s exact test, or the Cochran‒Armitage test. When the sample size was less than 10, Fisher’s exact test was employed. Correlations between continuous variables were assessed using Spearman’s correlation and linear regression analyses. On the basis of the genotyping results for each SNP, homozygotes for the allele more common in Tibetan highlanders were classified as adaptive types, whereas heterozygotes and homozygotes for the allele less common in Tibetan highlanders were classified as nonadaptive types. When we compared the variation in Hb concentrations with age between adaptive and nonadaptive types, we employed analysis of covariance (ANCOVA). A *p* value < 0.05 was considered statistically significant. The software used for each analysis was R (ver. 4.3.2) and R studio (ver. 2023.09.1 + 494).

## Results

### Prevalence of lifestyle diseases and polycythemia

Table 2 presents the prevalence of lifestyle diseases among participants based on participants meeting the criteria for each lifestyle disease. No statistically significant difference in age was observed between sexes (*p* = 0.15). Among the male participants, 29.5% (*n* = 23) met the criteria for obesity, whereas among female participants, 25.6% (*n* = 23) met the criteria for obesity. High blood pressure was observed in 25.6% (*n* = 20) of males and 16.7% (*n* = 15) of females, with a higher prevalence noted among males. Diabetes was detected in 4.2% (*n* = 3) of the males and 4.6% (*n* = 4) of the females. Hypoxemia was present in 25.6% (*n* = 20) of the males and 27.8% (*n* = 25) of the females, with a higher prevalence of hypoxemia observed among females. However, no statistically significant between-sex differences in these prevalences were found. Conversely, polycythemia was observed only in female participants, indicating a significant sex difference in polycythemia prevalence (*p* < 0.01).

**Table 2:** Prevalence of NCDs, hypoxia, and polycythemia by sex

Variables	Overall (*n* = 168)	Males (*n* = 78)	Females (*n* = 90)	*p* value
Age, years	46.7 ± 14.5	45.0 ± 13.1	48.2 ± 15.5	0.15
Obesity^a^	46 (27.4)	23 (29.5)	23 (25.6)	0.69
Hypertension^a^	35 (20.8)	20 (25.6)	15 (16.7)	0.22
Diabetes^b^	7 (4.4)	3 (4.2)	4 (4.6)	1.00
Hypoxia^a^	45 (26.8)	20 (25.6)	25 (27.8)	0.89
Polycythemia^c^	11 (6.5)	0 (0.0)	11 (12.2)	< 0.01

Sex differences in age were analyzed by Welch’s *t* test, and sex differences in prevalence were analyzed by the chi-square test or Fisher’s exact test

^a^*p* values indicate the results of the chi-square test

^b^HbA1c concentrations could not be measured in 6 males and 3 females because of blood viscosity or machine issues

^c^Hb concentrations could not be measured in 7 males because of severe deformation of their fingers

*NCD*s Non-communicable diseases.

### Associations between genotype frequencies and the prevalence of lifestyle diseases

**Fig. 1:**
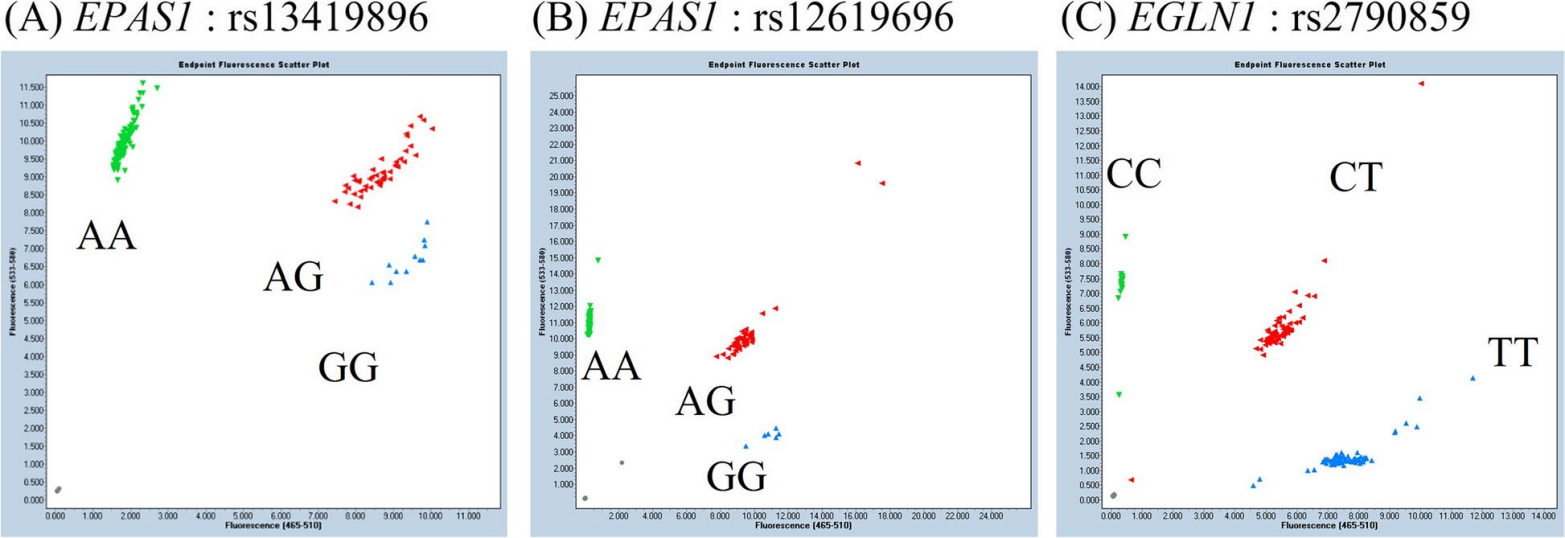
Result of real-time PCR for determining the genotype of each single-nucleotide polymorphism (SNP). Gray dots indicate negative controls

**Fig. 2:**
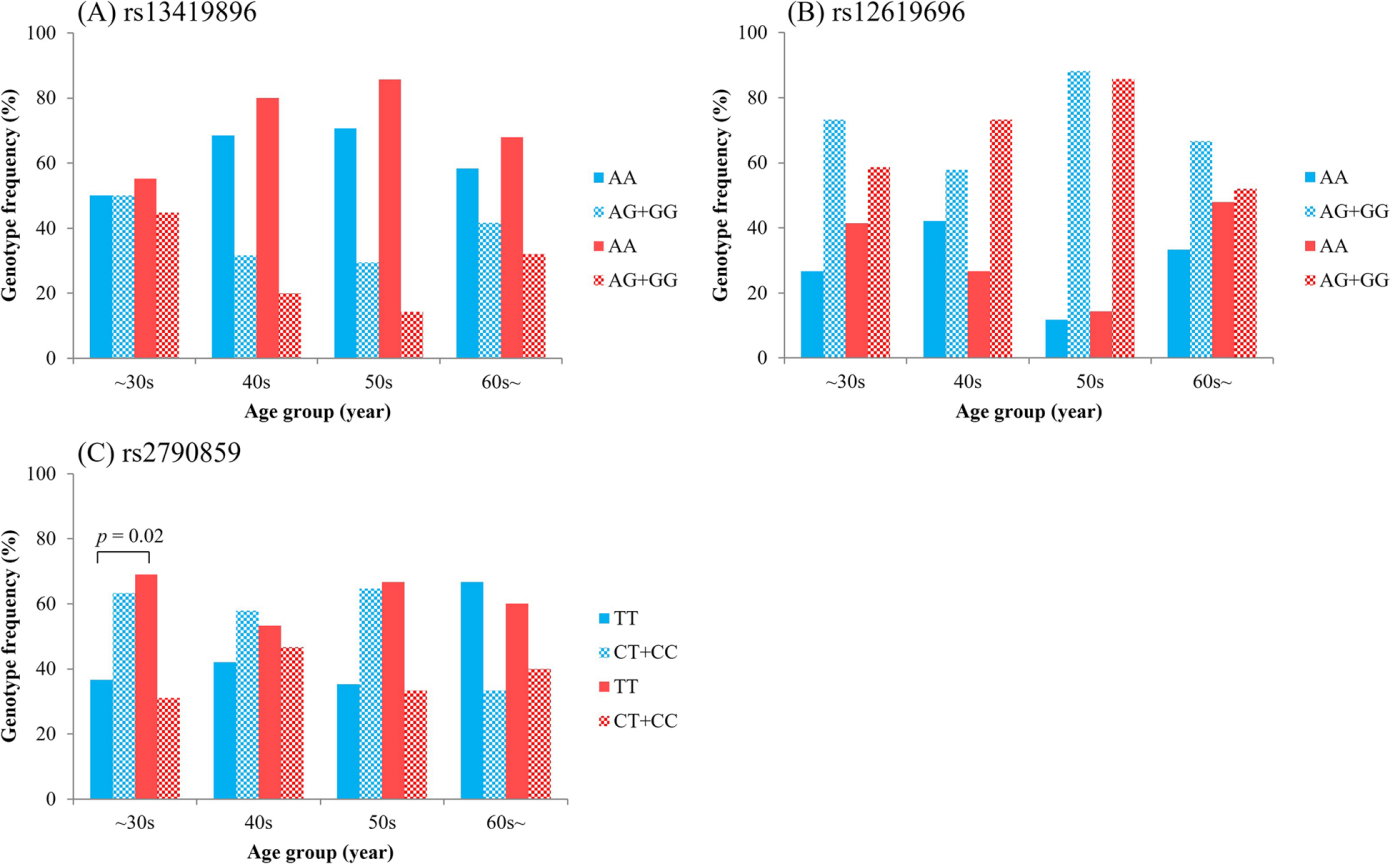
Genotype frequency by age group. Genotype frequencies by age group are shown. Blue indicates male data and red indicates female data in this figure. The *p* value indicates the results of the chi‐square test or Fisher’s exact test to compare the genotype frequency between males and females. A significant sex difference in rs2790859 genotype frequency was observed only in the 30 to 39 age group

The results of the PCR detection of the three SNPs targeted in this study are presented in Fig. 1 and Table 3. For rs13419896 and rs2790859, females presented a significantly greater proportion of adaptive genotypes and alleles than males did (rs13419896: genotype *p* < 0.05, allele *p* < 0.05; rs2790859: genotype *p* < 0.05, allele *p* < 0.05) (Table 3). Further comparison of genotype frequencies across different age groups revealed that females in all age groups had a greater proportion of participants adaptive genotypes except for the 40 to 49 age group for rs12619696 and the 60 and above age group for rs2790859 (Fig. 2). However, significant differences in genotype frequencies between males and females were observed only in the under-30 age group for rs2790859 (*p* < 0.05).

**Table 3:** Genotype frequency and allele frequency by sex

EPAS1	rs13419896	Overall			Males			Females			*p* value
	Genotype	AA	AG	GG	AA	AG	GG	AA	AG	GG	< 0.05
		110(65.5%)	47(30.0%)	11(6.5%)	47(60.3%)	22(28.2%)	9(11.5%)	63(70.0%)	25(27.8%)	2(2.2%)	
	Allele	A	G		A	G		A	G		< 0.05
		79.5%	20.5%		74.4%	25.6%		83.9%	16.1%		
	rs12619696	Overall			Males			Females			*p* value
	Genotype	AA	AG	GG	AA	AG	GG	AA	AG	GG	0.26
		53(31.5%)	109(64.9%)	6(3.6%)	22(28.2%)	52(66.7%)	4(5.1%)	31(34.4%)	57(63.3%)	2(2.2%)	
	Allele	A	G		A	G		A	G		0.45
		64.0%	36.0%		61.5%	38.5%		66.1%	33.9%		
EGLN1	rs2790859	Overall			Males			Females			*p* value
	Genotype	TT	CT	CC	TT	CT	CC	TT	CT	CC	< 0.05
		90(53.6%)	63(37.5%)	15(8.9%)	33(42.3%)	38(48.7%)	7(9.0%)	57(63.3%)	25(27.8%)	8(8.9%)	
	Allele	T	C		T	C		T	C		< 0.05
		72.3%	27.7%		66.7%	33.3%		77.2%	22.8%

The sex difference in genotype frequency was analyzed via the Cochran–Armitage test, and the sex difference in allele frequency was analyzed via the chi-square test.

Furthermore, we compared the prevalence of each disease across age groups between participants in the adaptive and nonadaptive genotype groups (Table 4). In females with rs2790859, the prevalence of polycythemia was significantly lower in the adaptive genotype group than in the nonadaptive genotype group (*p* < 0.01), whereas no significant associations were observed between the genotype frequencies of SNPs and other diseases.

**Table 4:** Comparison of the prevalence of NCDs and polycythemia between the adaptive group and nonadaptive group

Males	rs13419896			rs12619696			rs2790859		
	AA (*n* = 47)	AG + GG (*n* = 31)	*p* value	AA (*n* = 22)	AG + GG (*n* = 56)	*p* value	TT (*n* = 33)	CT + CC (*n* = 45)	*p* value
Age, years	45.4 ± 13.6	44.4 ± 12.6	0.74	44.4 ± 12.8	45.2 ± 13.4	0.80	47.0 ± 13.8	43.6 ± 12.6	0.27
Obesity	13 (27.7)	10 (32.3)	0.86^c^	6 (27.3)	17 (30.4)	1.00	9 (27.3)	14 (31.1)	0.80
Hypoxia	12 (25.5)	8 (25.8)	1.00	5 (22.7)	15 (26.8)	0.78	11 (33.3)	9 (20.0)	0.20
Hypertension	10 (21.3)	10 (32.3)	0.41^c^	5 (22.7)	15 (26.8)	0.78	10 (30.3)	10 (22.2)	0.59^c^
Diabetes^a^	3 (6.8)	0 (0.0)	0.29	0 (0.0)	3 (5.7)	0.57	0 (36.7)	3 (7.1)	0.27
Polycythemia^b^	–	–	–	–	–	–	–	–	–
Females	rs13419896			rs12619696			rs2790859		
	AA (*n* = 63)	AG + GG (*n* = 27)	*p* value	AA (*n* = 31)	AG + GG (*n* = 59)	*p* value	TT (*n* = 57)	CT + CC (*n* = 33)	*p* value
Age, years	49.3 ± 15.6	45.5 ± 15.3	0.29	48.9 ± 16.8	47.8 ± 14.9	0.77	48.1 ± 15.6	48.3 ± 15.6	0.96
Obesity	15 (23.8)	8 (29.6)	0.60	8 (25.8)	15 (25.4)	1.00	15 (26.3)	8 (24.2)	1.00
Hypoxia	19 (30.2)	6 (22.2)	0.61	6 (19.4)	19 (32.2)	0.23	16 (28.1)	9 (27.3)	1.00
Hypertension	12 (19.0)	3 (11.1)	0.54	7 (22.6)	8 (13.6)	0.33	11 (19.3)	4 (12.1)	0.56
Diabetes^a^	3 (4.9)	1 (3.8)	1.00	2 (6.9)	2 (3.4)	0.60	4 (7.1)	0 (0.0)	0.30
Polycythemia	9 (14.3)	2 (7.4)	0.49	2 (6.5)	9 (15.3)	0.32	2 (3.5)	9 (27.3)	< 0.01

The data in the table are presented as the means ± SDs or *n* (%). Welch’s *t* test was used for the comparison of age between the groups, and the chi-square test or Fisher's exact test was used for the comparison of the prevalence of each disease

^a^HbA1c concentration could not be measured for 6 male and 3 female participants

^b^Polycythemia was not found in males

^c^*p* values indicate the results of the chi-square test

*NCDs* non-communicable diseases

### Changes in Hb concentration with age stratified by genotype

**Fig. 3:**
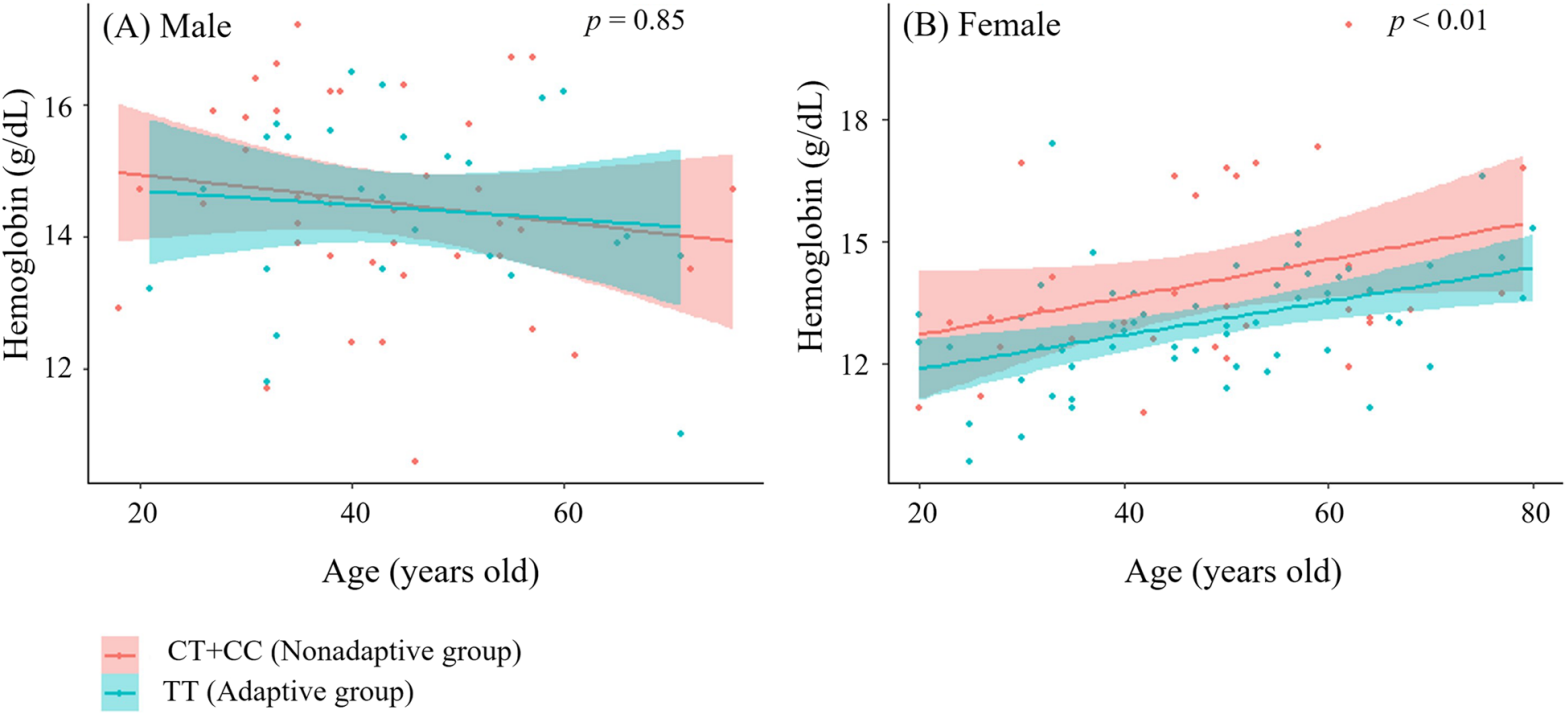
Relationship between hemoglobin concentration and age by rs2790859 genotype. Lines and ranges in the figure indicate approximation lines and ± standard deviation (SD). **A** The results of Spearman’s correlation analysis between hemoglobin concentration and age in participants with various genotypes were as follows: TT, *r* = − 0.0122 (linear regression analysis; *p* = 0.59) and CT + CC, *r* = − 0.1999 (linear regression analysis; *p* = 0.34. **B** The results of Spearman’s correlation analysis between hemoglobin concentration and age in participants with various genotypes were as follows: TT, *r* = 0.4468 (linear regression analysis;* p* < 0.01) and CT + CC, *r* = 0.3695 (linear regression analysis; *p* = 0.06). *p* values in this figure indicate the result of the analysis of covariance to compare differences in hemoglobin concentrations between the TT and CT + CC genotypes

We plotted the relationship between age and Hb concentrations stratified by the adaptive and nonadaptive genotypes of rs2790859 (Fig. 3). In males, Hb concentrations exhibited a consistent or gradual decline with increasing age, irrespective of the adaptive or nonadaptive genotype, and neither trend line demonstrated a statistically significant slope (*p* = 0.59, *p* = 0.34). In contrast, females with both the adaptive and nonadaptive genotypes presented a trend of increasing Hb concentrations with age (*p* < 0.01, *p* = 0.06). Furthermore, females with the adaptive genotype presented significantly lower Hb concentrations than those with the nonadaptive genotype (*p* < 0.01).

## Discussion

In this study, we aimed to investigate the genotype frequencies of polymorphisms previously found to be associated with human hypoxic adaptation and response and the relationship of these genotype frequencies with the prevalence of lifestyle diseases among Tsarang residents. This study revealed sex differences in the genotype frequencies of rs13419896 in *EPAS1* and rs2790859 in *EGLN1* among Tsarang residents. Carrying the rs2790859 SNP in *EGLN1*, which is involved in the hypoxia response, has been suggested as a risk factor for polycythemia in modern Tibetan highlanders living in Tsarang, indicating a potential association between genotype and disease susceptibility.

### Prevalence of polycythemia and lifestyle diseases among residents of Tsarang

Tibetan highlanders are thought to have acquired adaptive mechanisms to suppress the increase in Hb concentrations even in hypoxic environments [6]. However, in Tibetan highlanders from Sichuan Province, China, polycythemia was observed in 25.5% of males and 21.8% of females [27], whereas in Tibetan highlanders from Qinghai Province, polycythemia was observed in 31.6% of the population [28]. Reports suggest that the higher the altitude of their residence is, the higher the Hb concentrations among Tibetan highlanders [29]; however, the prevalence of polycythemia in Tsarang was relatively low, with no male participants classified as having polycythemia.

There is concern about the increasing prevalence of lifestyle diseases among modern Tibetan highlanders. In Tibetan highlanders inhabiting regions outside the study area, the prevalence of obesity (BMI > 25) exceeds 35%. Particularly among Tibetan highlanders living in Ladakh, India, 42.6% of males and 62.7% of females are classified as obese, whereas 47.9% of Tibetan highlanders living in China are reported to be obese [14, 30, 31]. Compared with the individuals evaluated in these previous studies, Tibetan highlanders living in Tsarang can still be considered to have a relatively low obesity rate. Furthermore, in Ladakh, 42.6% of males and 62.7% of females were found to have hypertension [30], indicating that the prevalence of hypertension was relatively low among residents of Tsarang. The prevalence of diabetes in Ladakh was found to be 25.5% among males and 8.8% among females [30], suggesting that the prevalence of diabetes in Tsarang is also lower than that in Tibetan highlanders living in other regions. Given that the prevalence of many lifestyle diseases among Tsarang residents is lower than that among Tibetan highlanders living in other regions, such as India and China, it is reasonable to conclude that dietary changes significant enough to promote the onset of these lifestyle diseases have not yet occurred in Tsarang.

However, with further development as a tourist destination and changes in dietary habits, there is a risk that the prevalence of lifestyle diseases among Tsarang residents may increase, as has occurred in Tibetan highlanders living in other regions. In previous studies comparing the relationship between diet and health risks among Han Chinese and Tibetan highlanders, Han Chinese individuals with high dietary diversity had lower blood glucose levels, whereas Tibetan highlanders with high dietary diversity tended to have higher blood glucose levels [32]. In other words, Tibetan highlanders consuming modern diets are at a greater risk of increased blood glucose levels because these diets are rich in nutrients and calories. It is necessary to continue observing lifestyle disease risk among Tsarang residents from both dietary changes and genotype perspectives.

### Sex differences in genotype frequencies among Tsarang residents

Significant sex differences in genotype and allele frequencies were observed for *EPAS1* rs13419896 and *EGLN1* rs2790859, with a greater proportion of males carrying nonadaptive gene variants. In theory, since a father's gametes equally carry either an X chromosome or a Y chromosome [33], alleles of genes located on autosomes should be inherited equally by the next generation regardless of the child's sex. However, there have been reports of SNPs located on autosomes that are associated with the risk of autoimmune diseases and exhibit sex differences in allele frequencies [34]. The sex differences in the genotype frequencies of *EPAS1* and *EGLN1* found in this study must be further investigated from both genomic and epigenomic perspectives to determine whether they influence sex-specific survival strategies in hypoxic environments. On the other hand, Tsarang is located in the Mustang District, which has a polyandrous marriage culture. In recent years, workers from outside have come to the area for agricultural work and the construction of roads and bridges [35]. Conversely, it has been reported that people from Mustang migrate to warmer regions during the winter and foreign countries to earn income from sources other than agriculture [36]. Investigating the factors contributing to sex differences in genotype frequencies among Tsarang residents from various perspectives, including the potential genetic flow due to marriage and migration for work, is important.

### Associations between polymorphisms and the onset of polycythemia and lifestyle diseases

It has been suggested that the hypoxic adaptation mechanisms acquired by Tibetan highlanders are vulnerable to aging and obesity. The onset of lifestyle diseases due to recent lifestyle changes is believed to have disrupted the unique Hb concentration suppression mechanisms of Tibetan highlanders, leading to an increase in the prevalence of polycythemia among this population [14]. While the factors mentioned above may contribute to the onset of polycythemia, the results of this study showed that Tsarang females with the nonadaptive rs2790859 variant had a significantly greater prevalence of polycythemia than females with the adaptive variant did. Therefore, in other regions where the proportion of individuals with nonadaptive genotypes and their descendants has increased, Tibetan highlanders may be more prone to developing polycythemia. Previous studies have also suggested that differences in the prevalence of chronic mountain sickness between Han Chinese and Tibetan highlanders are due to genetic differences [37]. Additionally, it has been reported that the risk of polycythemia varies among Tibetan highlanders depending on their genotype [18]. In contrast, this study confirmed that Hb concentrations increased with age in Tsarang women regardless of the genotype of the three SNPs analyzed. This finding indicates that although the SNPs analyzed in this study are associated with the risk of developing polycythemia, they are not related to the age-related increase in Hb concentration specific to Tsarang females. Future research targeting other genes and environmental factors is expected to shed light on the causes of this unique phenomenon.

The people of Mustang, referred to as the Loba, are recognized as Tibetan highlanders, similar to the highland inhabitants of China and India, who have undergone extensive genetic evaluations. However, the findings from comparative studies of the genomes of ancient human bones found in various parts of the Tibetan Plateau suggest that humans migrated to the Tibetan Plateau 40,000 years ago, and another group entered the plateau 10,000 years ago. Furthermore, regional characteristics of the genomes of Tibetan highlanders have been identified, with genetic distances increasing as the geographic distance from Mustang increases [38]. Owing to the genetic diversity among Tibetan highlanders, the findings of this study suggest that *EPAS1* genotypes may not be associated with Hb concentrations, which is different from the results of previous studies in which the genomes of Tibetan highlanders from other regions were analyzed. The exploration of hypoxic adaptation and disease susceptibility among Mustang residents from various perspectives beyond the framework of Tibetan highlanders is necessary.

## Limitations

This study focused on the village of Tsarang within the former Mustang Kingdom and therefore evaluated a unique population that has not experienced extensive intermixing, such as Tibetan highlander populations in China and India. However, a limitation of this study is the small sample size due to its focus on only one settlement. Furthermore, this study did not evaluate lifestyle disease risk through the analysis of SNPs that were not a focus of the study, gene expression levels, methylation levels, or other epigenetic factors. Future analyses are needed to elucidate the hypoxic adaptations of modern Tibetan highlanders and the various mechanisms that induce disease susceptibility.

## Conclusions

This study revealed sex differences in the genotype frequencies of genes involved in the hypoxia response among Tsarang residents. Additionally, the rs2790859 polymorphism was suggested to potentially contribute to the recent onset of polycythemia in this area. In the future, it is important to be vigilant, as intermixing with other populations in Tsarang could lead to an increase in non-Tibetan genotypes. The findings of this study could raise awareness about polycythemia as a modern health issue.

## Data Availability

The datasets used and/or analyzed during the current study are available from the corresponding author upon reasonable request.

## Abbreviations

BMI: Body mass index
DBP: Diastolic blood pressure
EAS: East Asian
*EGLN1*: Egl-9 family hypoxia-inducible factor 1
*EPAS1*: Endothelial per-ARNT-sim domain protein 1
Hb: Hemoglobin
NCDs: Noncommunicable diseases
NO: Nitric oxide
SAS: South Asian
SBP: Systolic blood pressure
SD: Standard deviation
SNP: Single-nucleotide polymorphism
SpO_2_: Saturation of peripheral oxygen
